# ASK1 controls spindle orientation and positioning by phosphorylating EB1 and stabilizing astral microtubules

**DOI:** 10.1038/celldisc.2016.33

**Published:** 2016-10-04

**Authors:** Youguang Luo, Jie Ran, Songbo Xie, Yunfan Yang, Jie Chen, Shanshan Li, Wenqing Shui, Dengwen Li, Min Liu, Jun Zhou

**Affiliations:** 1 Institute of Biomedical Sciences, College of Life Sciences, Key Laboratory of Animal Resistance Biology of Shandong Province, Key Laboratory of Molecular and Nano Probes of the Ministry of Education, Shandong Normal University, Jinan, China; 2 State Key Laboratory of Medicinal Chemical Biology, Key Laboratory of Protein Science of Tianjin, Key Laboratory of Bioactive Materials of the Ministry of Education, Department of Genetics and Cell Biology, College of Life Sciences, Nankai University, Tianjin, China

**Keywords:** apoptosis signal-regulating kinase 1, astral microtubule, end-binding protein 1, phosphorylation, spindle orientation, spindle positioning

## Abstract

Orientation and positioning of the mitotic spindle are involved in dictating cell division axis and cleavage site, and play important roles in cell fate determination and tissue morphogenesis. However, how spindle movement is controlled to achieve a defined alignment within the dividing cell is not fully understood. Here, we describe an unexpected role for apoptosis signal-regulating kinase 1 (ASK1) in regulating spindle behavior. We find that ASK1 is required for proper mitotic progression and daughter cell adhesion to the substratum. ASK1 interacts with end-binding protein 1 (EB1) and phosphorylates EB1 at serine 40, threonine 154 and threonine 206, enhancing its binding to the plus ends of astral microtubules. Consequently, astral microtubules are stabilized and therefore capable of mediating spindle interaction with the cell cortex, a requirement for spindle movement. These findings reveal a previously undiscovered function of ASK1 in cell division by regulating spindle orientation and positioning, and point to the importance of protein phosphorylation in the regulation of spindle behavior.

## Introduction

Cell division is a fine-tuned process that is spatiotemporally controlled by the mitotic spindle, a dynamic structure composed of microtubules and microtubule-associated proteins. In addition to the orchestration of chromosome movement and segregation, the spindle is critically involved in determining cell division axis and cleavage site, which depend on proper orientation and positioning of the spindle relative to the cell cortex [1, 2]. Spindle orientation and positioning are of paramount importance in cell fate specification and tissue organization during development as well as in adult life. Mutations causing human diseases, such as neurological disorders and cancer, have been associated with spindle orientation/positioning defects, although the causal relationship between these diseases and spindle orientation/positioning defects remains unclear [3].

The interaction of astral microtubules with the cell cortex lies at the core of the mechanism for spindle orientation and positioning [1, 4]. There is accumulating evidence that alteration of proteins involved in astral microtubule stability, such as end-binding protein 1 (EB1) and many other microtubule plus end tracking proteins (+TIPs), often results in spindle orientation/positioning defects [5–8]. In addition to binding microtubules directly, EB1 can recruit many other +TIPs to microtubule plus ends [9]. It is believed that these +TIPs act in concert to regulate the stability of astral microtubules and the interaction of astral microtubules with the cell cortex [7, 10]. However, how the functions of +TIPs in spindle orientation/positioning are regulated and whether the regulation is linked to their effects on astral microtubules remain largely unknown. In this study, we offer the first evidence that apoptosis signal-regulating kinase 1 (ASK1), a serine/threonine kinase of the mitogen-activated protein kinase kinase kinase family critical for stress and immune responses [11], participates in spindle orientation and positioning by phosphorylating EB1 and stabilizing astral microtubules.

## Results

### ASK1 localizes to spindle poles and is important for proper cell division

**Figure 1:**
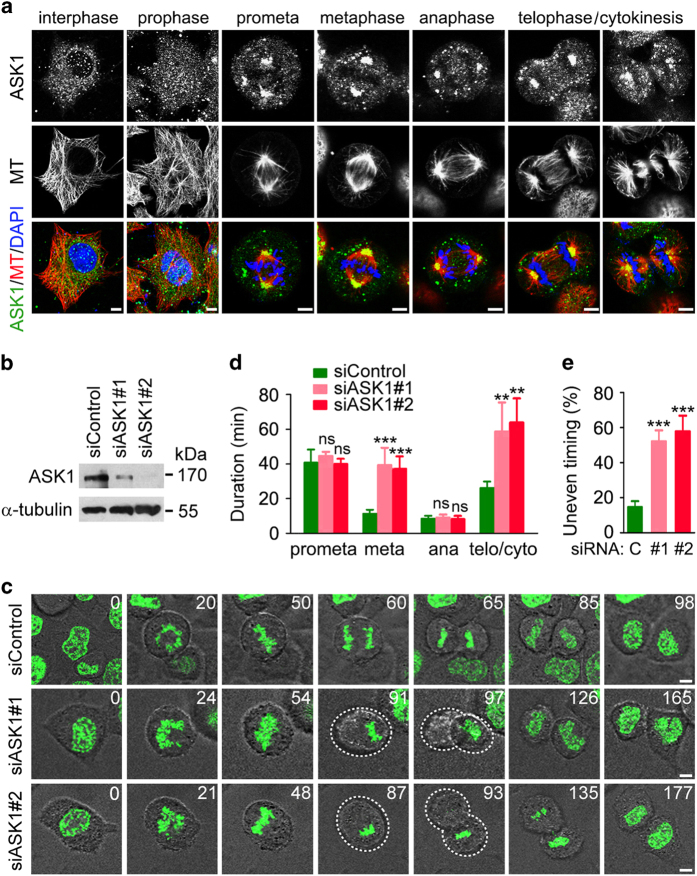
ASK1 localizes to spindle poles and is important for proper cell division. (**a**) Immunofluorescence images of HeLa cells stained with anti-ASK1 and anti-α-tubulin antibodies and DAPI. Scale bars, 5 μm. (**b**) Immunoblots for ASK1 and α-tubulin expression in control and ASK1 siRNA-treated HeLa-H2B cells. (**c**) Time-lapse images showing prolonged metaphase and telophase/cytokinesis and uneven timing of daughter cell adhesion to the substratum in ASK1 siRNA-treated HeLa-H2B cells. Dashed lines indicate daughter cells that are not in the same focal plane. The unit for time is minute. (**d**) Duration of different mitotic phases in cells treated as in (**c**). *n*=10 cells per group. (**e**) Quantification of cell divisions with uneven timing of daughter cell adhesion to the substratum in cells treated as in (**c**). *n*=10 cells per group. Scale bars, 5 μm. Experiments were performed three times. Values are mean±s.e.m. **P<0.01, ***P<0.001; NS, not significant.

In an attempt to identify novel functions of ASK1, we analyzed its localization in HeLa cells. Immunofluorescence microscopy showed a diffuse distribution for ASK1 in interphase and prophase cells (Figure 1a). Interestingly, ASK1 was enriched at spindle poles from prometaphase to cytokinesis (Figure 1a). The localization of ASK1 at spindle poles was abolished in cells transfected with ASK1 siRNAs (Supplementary Figure S1). A similar localization pattern was observed for HA-tagged ASK1 (Supplementary Figure S2), suggesting a potential role for this protein in mitotic progression.

To test this possibility, we knocked down ASK1 expression by using two different siRNAs (Figure 1b). Time-lapse microscopy of HeLa cells stably expressing YFP-tagged histone 2B (hereinafter named HeLa-H2B) revealed that knockdown of ASK1 caused a delay in mitotic progression due to the prolongation of metaphase and telophase/cytokinesis (Figure 1c and d). ASK1 knockdown cells also exhibited uneven timing of daughter cell adhesion to the substratum (Figure 1c and e), suggesting that ASK1 might be required for proper orientation and positioning of the mitotic spindle.

### Depletion of ASK1 impairs spindle orientation and positioning

**Figure 2:**
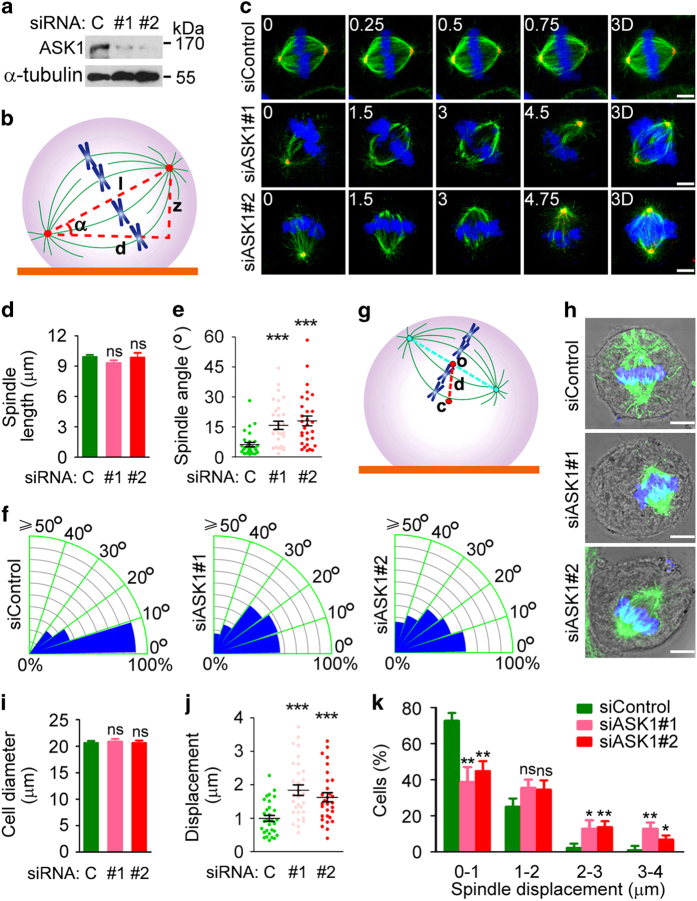
Depletion of ASK1 impairs spindle orientation and positioning. (**a**) Immunoblots showing the expression of ASK1 and α-tubulin in control or ASK1 siRNA-treated HeLa cells. (**b**) Scheme for spindle angle (*α*) measurement. (**c**–**f**) Immunofluorescence images (**c**), spindle length (**d**), spindle angle (**e**) and spindle angle distribution (**f**) of metaphase HeLa cells transfected with control or ASK1 siRNAs and stained with anti-α-tubulin (green) and anti-γ-tubulin (red) antibodies and DAPI (blue). The position of the z stage is indicated in micrometers; 3D, *xy* projection. *n*=30 cells per group. Scale bars, 5 μm. (**g**) Scheme for spindle displacement distance (*d*) measurement. (**h**–**k**) Immunofluorescence/phase-contrast images (**h**), cell diameter (**i**), spindle displacement distance (**j**) and spindle displacement distance distribution (**k**) of control and ASK1 siRNA-treated metaphase HeLa cells stained with anti-α-tubulin antibody (green) and DAPI (blue). *n*=30 cells per group. Scale bars, 5 μm. Experiments were performed three times. Values are mean±s.e.m. *P<0.05, **P<0.01, ***P<0.001; NS, not significant.

We examined the potential role of ASK1 in spindle orientation by measuring the angle between the spindle axis and the substratum (referred to as spindle angle) in ASK1-depleted HeLa cells (Figure 2a and b). Immunofluorescence microscopy revealed that knockdown of ASK1 did not significantly influence the overall morphology or length of the spindle (Figure 2c and d). However, the average spindle angle was increased from less than 5° to over 15° upon the loss of ASK1 (Figure 2c and e). In addition, ASK1 siRNAs remarkably broadened the distribution of spindle angles (Figure 2f).

We then sought to investigate whether the loss of ASK1 affects spindle positioning, by measuring the distance between the spindle center and the cell center (referred to as spindle displacement distance) (Figure 2g). We found that ASK1 depletion did not obviously affect cell diameter, but resulted in a significant increase in spindle displacement distance (Figure 2h–k), indicating spindle positioning defects. Collectively, the above results reveal an important function for ASK1 in the control of spindle orientation and positioning.

### ASK1 activity is required for spindle orientation/positioning and astral microtubule stability

**Figure 3:**
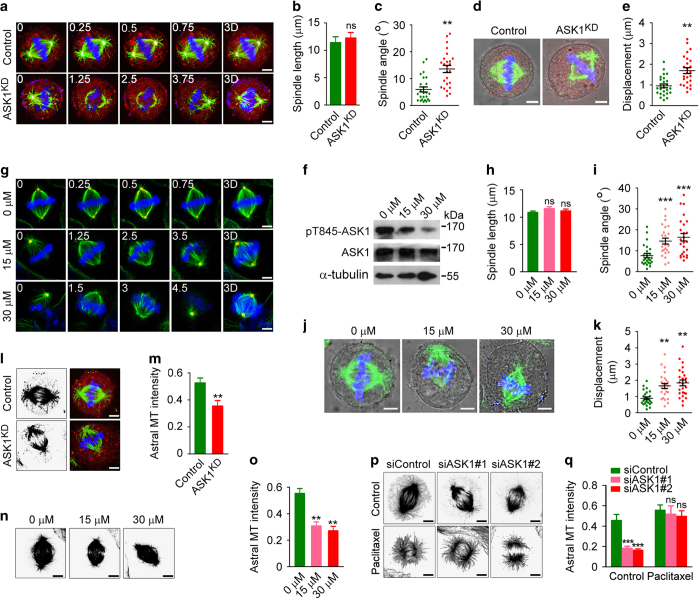
ASK1 activity is required for spindle orientation/positioning and astral microtubule stability. (**a**–**c**) Immunofluorescence images (**a**), spindle length (**b**) and spindle angle (**c**) of metaphase HeLa cells transfected with mCherry (red) in the absence or presence of ASK1^KD^ and stained with anti-α-tubulin (green) and anti-γ-tubulin (purple) antibodies and DAPI (blue). The position of the z stage is indicated in micrometers; 3D, *xy* projection. *n*=25 cells per group. Scale bars, 5 μm. (**d**, **e**) Immunofluorescence/phase-contrast images (**d**) and spindle displacement distance (**e**) of metaphase HeLa cells transfected as in (**a**) and stained with anti-α-tubulin antibody (green) and DAPI (blue). Red, mCherry. *n*=25 cells per group. Scale bars, 5 μm. (**f**) Immunoblots showing the levels of ASK1 phosphorylated at threonine 845 (pT845-ASK1), ASK1 and α-tubulin in HeLa cells treated with 0, 15 or 30 μm NQDI-1. (**g**–**i**) Immunofluorescence images (**g**), spindle length (**h**) and spindle angle (**i**) of metaphase HeLa cells treated with NQDI-1, and stained as in (**a**). *n*=25 cells per group. Scale bars, 5 μm. (**j**, **k**) Immunofluorescence/phase-contrast images (**j**) and spindle displacement distance (**k**) of metaphase HeLa cells treated with NQDI-1 and stained as in (**d**). *n*=25 cells per group. Scale bars, 5 μm. (**l**, **m**) Immunofluorescence images (**l**) and astral microtubule intensity (**m**) of metaphase HeLa cells transfected as in (**a**) and stained with anti-α-tubulin antibody (green) and DAPI (blue). Red, mCherry. *n*=30 cells per group. Scale bars, 5 μm. (**n**, **o**) Immunofluorescence images (**n**) and astral microtubule intensity (**o**) of metaphase HeLa cells treated with NQDI-1 and stained with anti-α-tubulin antibody. *n*=25 cells per group. Scale bars, 5 μm. (**p**, **q**) Immunofluorescence images (**p**) and astral microtubule intensity (**q**) of metaphase HeLa cells transfected with control or ASK1 siRNAs, treated with control (DMSO) or 0.5 μm paclitaxel, and stained with anti-α-tubulin antibody. *n*=25 cells per group. Scale bars, 5 μm. Experiments were performed three times. Values are mean±s.e.m. **P<0.01, ***P<0.001; NS, not significant.

To determine whether the kinase activity of ASK1 is necessary for its role in spindle orientation and positioning, HeLa cells were transfected with a kinase-dead, dominant-negative mutant of ASK1 (ASK1^KD^), in which lysine 709 was substituted by arginine [12]. mCherry fluorescent protein was cotransfected to detect transfected cells. Consistent with the data obtained by siRNA experiments, the ASK1^KD^ mutant did not obviously influence spindle length, but dramatically increased spindle angle (Figure 3a–c) and spindle displacement distance (Figure 3d and e). In addition, inhibition of ASK1 activity by NQDI-1, a specific inhibitor of ASK1 [13], led to spindle orientation/positioning defect similar to that caused by ASK1^KD^ (Figure 3f–k). These data demonstrate a critical requirement for ASK1 activity in spindle orientation and positioning.

To gain mechanistic insight into the regulation of spindle behavior by ASK1, we examined the morphology of spindle microtubules. By immunofluorescence microscopy, we found that both ASK1^KD^ and NQDI-1 resulted in a significant reduction of astral microtubule intensity (Figure 3l–o). Similarly, siRNA-mediated knockdown of ASK1 expression dramatically decreased the intensity of astral microtubules, and this effect was rescued by the microtubule-stabilizing agent paclitaxel (Figure 3p and q). These results reveal that ASK1 regulates astral microtubule stability in a kinase activity-dependent manner.

### ASK1 interacts with EB1 both in cells and *in vitro*

**Figure 4:**
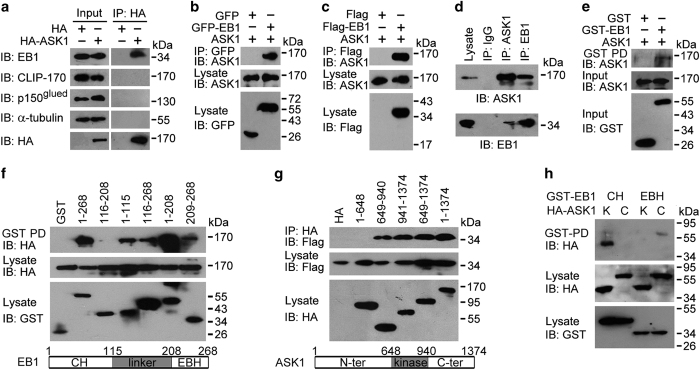
ASK1 interacts with EB1 both in cells and *in vitro*. (**a**) Immunoprecipitation (IP) and immunoblotting (IB) showing that HA-ASK1 interacts with endogenous EB1, but not CLIP-170, p150^glued^, or α-tubulin in 293 T cells. (**b**–**d**) Immunoprecipitation and immunoblotting showing that endogenous ASK1 interacts with GFP-EB1 (**b**), Flag-EB1 (**c**) and endogenous EB1 (**d**) in 293 T cells. (**e**) GST pulldown (PD) and immunblotting showing that *in vitro*-translated ASK1 interacts with bacterially purified GST-EB1, but not GST. (**f**, **g**) Characterization of the domains mediating the interaction between ASK1 and EB1 in cells transfected with HA-ASK1 and different forms of EB1 tagged with GST (**f**), or Flag-EB1 and different forms of ASK1 tagged with HA (**g**). Schematic diagrams of the CH domain, linker region and EBH domain of EB1 (**f**), and the N-terminal region, kinase domain and C-terminal region of ASK1 (**g**) are shown below the blots. (**h**) GST pulldown and immunblotting showing that the CH domain of EB1 interacts with the kinase (K) domain of ASK1 and that the EBH domain of EB1 interacts with the C-terminal (C) region of ASK1 in 293 T cells.

We then investigated how ASK1 regulates astral microtubules. Our previous yeast two-hybrid initiative, using HeLa cell cDNA library with EB1 as bait, identified two positive cDNAs encoding different fragments of ASK1, in addition to the identification of the mitotic kinase Aurora-B [14]. Immunoprecipitation revealed that HA-ASK1 interacted with endogenous EB1, but not two other +TIPs, cytoplasmic linker protein 170 (CLIP-170) and p150^glued^, or tubulin (Figure 4a). We also found that endogenous ASK1 was able to interact with GFP-EB1, Flag-EB1 and endogenous EB1 (Figure 4b–d). Immunofluorescence microscopy revealed a colocalization of ASK1 and EB1 at spindle poles (Supplementary Figure S3). GST pulldown assay using bacterially purified GST-EB1 and *in vitro* translated ASK1 further showed a direct interaction between these two proteins (Figure 4e).

By using a series of truncated EB1, we found that the calponin-homology (CH) domain (1–115) and the EB-homology domain (EBH) (209–268) of EB1, but not its linker region (116–208), were sufficient for its interaction with ASK1 (Figure 4f). In addition, the kinase domain (649–940) and the C-terminal region (941–1374) of ASK1, but not its N-terminal region, were sufficient for its interaction with EB1 (Figure 4g). Further analysis showed that the CH domain of EB1 interacted strongly with the kinase domain of ASK1 and that the EBH domain of EB1 interacted weakly with the C-terminal region of ASK1 (Figure 4h).

### ASK1 phosphorylates EB1 at S40, T154 and T206

**Figure 5:**
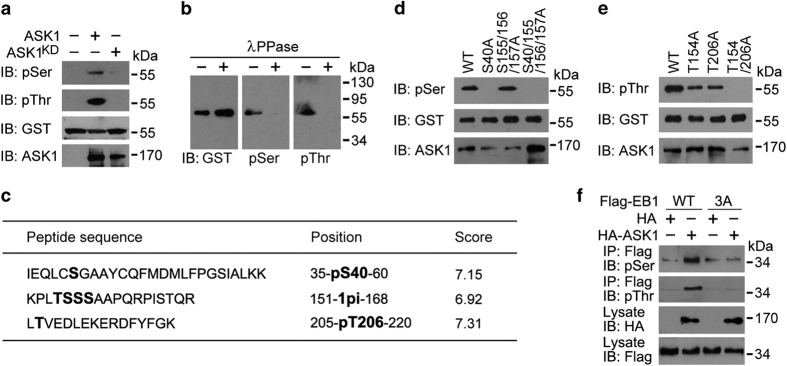
ASK1 phosphorylates EB1 at S40, T154 and T206. (**a**) Kinase assays were performed by using ASK1 or ASK1^KD^ immunoprecipitate from 293 T cells, with bacterially purified GST-EB1 as a substrate. The reaction mixture was then subjected to immunoblotting with phosphoserine (pSer) and phosphothreonine (pThr) antibodies. (**b**) Kinase assays were performed as in (**a**), and GST-EB1 was pulled down from the reaction mixture and treated with λPPase. Immunoblotting was then performed with the indicated antibodies. (**c**) Kinase assays were performed as in (**a**), and EB1 phosphorylation sites were identified by mass spectrometry. (**d**, **e**) Kinase assays were performed by using ASK1 immunoprecipitate and bacterially purified GST-EB1 wild-type (WT) or mutants. The reaction mixture was then subjected to immunoblotting with pSer (**d**) and pThr (**e**) antibodies. (**f**) Immunoprecipitation and immunoblotting showing the level of EB1 phosphorylation in 293 T cells transfected with Flag-EB1-WT or -3A, together with HA or HA-ASK1. 3A, S40/T154/T206A.

We then analyzed whether EB1 undergoes phosphorylation by ASK1. To test this possibility, we performed *in vitro* kinase assays using ASK1 or ASK1^KD^ immunoprecipitate from 293 T cells and bacterially purified GST-EB1. Immunoblotting of the reaction mixture with phosphoserine and phosphothreonine antibodies revealed that EB1 was phosphorylated at both serine and threonine residues by ASK1 but not ASK1^KD^ (Figure 5a). Furthermore, ASK1-induced EB1 phosphorylation at serine and threonine residues was abrogated when GST-EB1 pulled down from the above reaction mixture was treated with λPPase (Figure 5b), confirming EB1 phosphorylation by ASK1.

To identify the residues of EB1 phosphorylated by ASK1, GST-EB1 pulled down from the above reaction mixture was subjected to SDS-PAGE and in-gel tryptic digestion. Subsequent mass spectrometric analysis of the peptides identified six potential phosphorylation sites, among which S40 is located in the CH domain, T154, S155, S156 and S157 in the linker region, and T206 in the linker region adjacent to the EBH domain (Figure 5c and Supplementary Figure S4). For the consecutive linker-region residues T154, S155, S156 and S157, tandem mass spectrometric analysis indicated that only one of the residues was phosphorylated, but the exact phosphorylation site could not be unambiguously assigned (Figure 5c and Supplementary Figure S4).

To further characterize the phosphorylation sites of EB1, we compared by *in vitro* kinase assays the level of phosphorylation of different EB1 mutants. While EB1 serine phosphorylation by ASK1 was not affected by mutation of S155, S156 and S157 to alanines, it was completely lost when S40 alone was mutated to alanine or when S40, S155, S156 and S157 were all mutated to alanines (Figure 5d). In addition, EB1 threonine phosphorylation by ASK1 was partially reduced by mutation of either T154 or T206 to alanine, and was completely lost when both T154 and T206 were mutated to alanines (Figure 5e). These results thus reveal S40, T154 and T206 as the residues of EB1 phosphorylated by ASK1.

We then investigated whether ASK1 phosphorylates EB1 at these three residues in cells. We transfected 293 T cells with HA or HA-ASK1, together with Flag-tagged wild-type EB1 or the phospho-deficient 3A mutant (mutation of S40, T154 and T206 to alanines). As shown in Figure 5f, overexpression of ASK1 in 293 T cells remarkably increased serine/threonine phosphorylation of wild-type EB1, but not the 3A mutant. By using an antibody against EB1 phosphorylated at T206, we further found that overexpression of wild-type ASK1 significantly enhanced EB1 phosphorylation in 293 T cells and that the kinase-dead, dominant-negative mutant of ASK1 (ASK1^KD^) had an opposite effect (Supplementary Figure S5). We also found that the ASK1 inhibitor NQDI-1 could block the ability of ASK1 to romote EB1 phosphorylation at T206 (Supplementary Figure S5).

### Actions of ASK1 in regulating astral microtubule stability and spindle orientation/positioning are mediated by its phosphorylation of EB1

**Figure 6:**
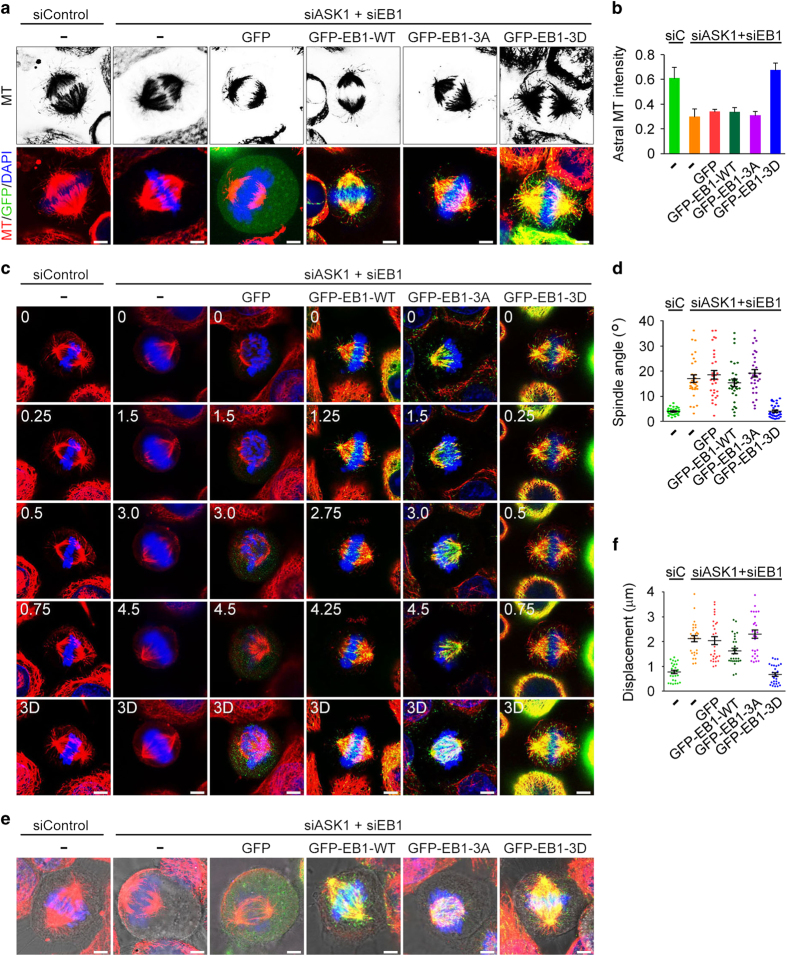
Actions of ASK1 in regulating astral microtubule stability and spindle orientation/positioning are mediated by its phosphorylation of EB1. (**a**, **b**) Immunofluorescence images (**a**) and astral microtubule intensity (**b**) of metaphase HeLa cells transfected with ASK1 and EB1 siRNAs and GFP or GFP-EB1-WT, -3A, or -3D and stained with anti-α-tubulin antibody and DAPI. *n*=30 cells per group. Scale bars, 5 μm. (**c**, **d**) Immunofluorescence images (**c**) and spindle angle (**d**) of metaphase HeLa cells transfected as in (**a**) and stained with anti-α-tubulin antibody (red) and DAPI (blue). *n*=30 cells per group. Scale bars, 8 μm. (**e**, **f**) Immunofluorescence/phase-contrast images (**e**) and spindle displacement distance (**f**) of metaphase HeLa cells transfected as in (**a**) and stained with anti-α-tubulin antibody (red) and DAPI (blue). *n*=30 cells per group. Scale bars, 5 μm. 3A, S40/T154/T206A; 3D, S40/T154/T206D. Experiments were performed three times. Values are mean±s.e.m.

We then studied whether EB1 phosphorylation is involved in the action of ASK1 in regulating astral microtubule stability, by using the phospho-deficient 3A mutant and the phospho-mimic 3D mutant (mutation of S40, T154 and T206 to aspartic acids) of EB1. Immunofluorescence microscopy revealed that ASK1/EB1 siRNA-induced reduction of astral microtubules was rescued by the 3D mutant, but not by wild-type EB1 or the 3A mutant (Figure 6a and b). In addition, the 3D mutant, but not wild-type EB1 or the 3A mutant, was able to restore ASK1/EB1 siRNA-induced spindle orientation/positioning defects, as evidenced by the decrease of spindle angles and spindle displacement distance (Figure 6c–f). These results suggest that the actions of ASK1 in regulating astral microtubule stability and spindle orientation/positioning are mediated by its phosphorylation of EB1.

### EB1 phosphorylation enhances its binding to the plus ends of astral microtubules

**Figure 7:**
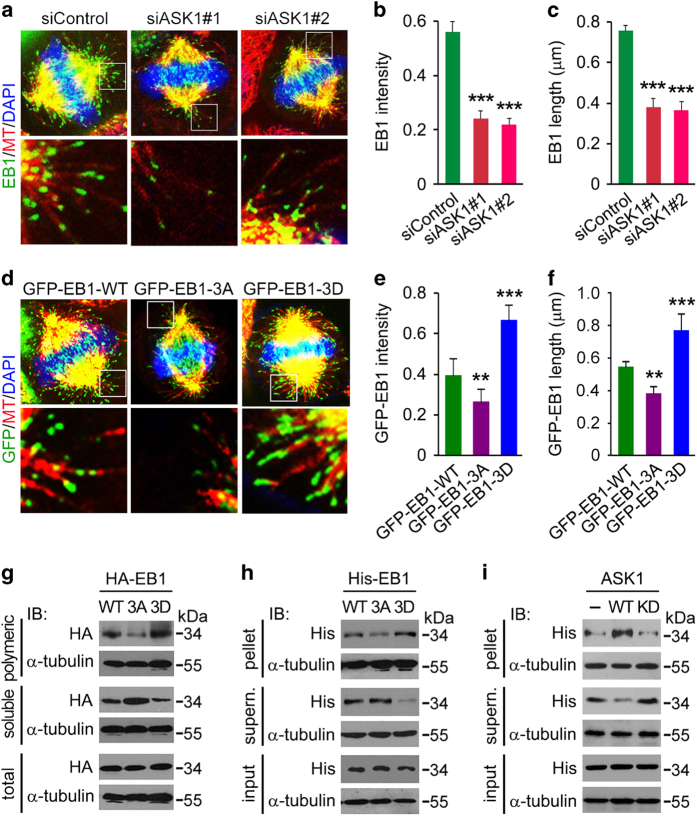
EB1 phosphorylation enhances its binding to the plus ends of astral microtubules. (**a**–**c**) Immunofluorescence images (**a**), intensity (**b**) and length (**c**) of EB1 at the plus ends of astral microtubules in metaphase HeLa cells transfected with control or ASK1 siRNAs and stained with DAPI and antibodies against α-tubulin and EB1. The intensity and length of EB1 comets were analyzed with Image J. *n*=100 comets from 10 cells. (**d**–**f**) Immunofluorescence images (**d**), intensity (**e**) and length (**f**) of GFP-EB1 at the plus ends of astral microtubules in metaphase HeLa cells transfected with EB1 siRNA and GFP-EB1-WT, -3A or -3D and stained with anti-α-tubulin antibody and DAPI. The intensity and length of EB1 comets were analyzed with Image J. *n*=100 comets from 10 cells. (**g**) 293 T cells were transfected with HA-EB1-WT, -3A or -3D. Cell extracts containing polymeric and soluble tubulin fractions were then prepared and analyzed by immunoblotting. Please see ‘Materials and Methods’ for experimental details. (**h**) Bacterially purified His-EB1-WT, -3A or -3D was incubated with preassembled microtubules for 30 min. Microtubules were then pelleted by centrifugation, and proteins in the pellet and supernatant fractions were analyzed by immunoblotting. Please see ‘Materials and Methods’ for experimental details. (**i**) Kinase assays were performed by using ASK1 or ASK1^KD^ immunoprecipitate from 293 T cells, with bacterially purified His-EB1 as a substrate. His-EB1 pulled down from the above mixture was then incubated with preassembled microtubules for 30 min. Microtubules were then pelleted by centrifugation, and proteins in the pellet and supernatant fractions were analyzed by immunoblotting. Please see ‘Materials and Methods’ for experimental details. Experiments were performed three times. Values are mean±s.e.m. **P<0.01, ***P<0.001.

To understand how EB1 phosphorylation contributes to astral microtubule stability, we examined its localization on astral microtubules. By immunofluorescence microscopy, we found that depletion of ASK1 significantly decreased the intensity and length of EB1 comets at the plus ends of astral microtubules in HeLa cells (Figure 7a–c). In addition, the localization of the phospho-deficient 3A mutant of EB1 at the plus ends of astral microtubules was weaker than wild-type EB1, whereas the localization of the phospho-mimic 3D mutant of EB1 was much stronger than wild-type EB1 (Figure 7d–f).

To corroborate the above findings, we transfected cells with wild type or mutant EB1 and then prepared polymeric and soluble tubulin fractions. We found that less 3A and more 3D were present in the polymeric fraction and more 3A and less 3D in the soluble fraction, as compared with wild-type EB1 (Figure 7g), suggesting that EB1 phosphorylation at S40, T154 and T206 promotes its binding to microtubules. By microtubule cosedimentation assays with purified proteins, we further found that wild type and mutant EB1 also differed in their microtubule-binding abilities *in vitro*, with the 3D mutant binding microtubules better than wild type and the 3A mutant (Figure 7h). To further investigate whether EB1 phosphorylation increases its microtubule-binding ability *in vitro*, we performed kinase assays using ASK1 immunoprecipitated from 293 T cells, with bacterially purified EB1 as a substrate, and then incubated EB1 from the above reaction mixture with preassembled microtubules. By microtubule cosedimentation assays, we found that ASK1-mediated EB1 phosphorylation greatly enhanced its binding to microtubules, as compared with addition of the ASK1^KD^ immunoprecipitate in the kinase assay or no kinase added (Figure 7i). Taken together, these data indicate that EB1 phosphorylation enhances its binding to the plus ends of astral microtubules.

## Discussion

ASK1 functions as a mitogen-activated protein kinase kinase kinase that activates the c-Jun N-terminal kinase and p38 signaling cascades in response to diverse environmental stresses, such as oxidative stress, endoplasmic reticulum stress and calcium overload [11]. In addition, ASK1 is an evolutionarily conserved component of innate immune response pathways induced by receptor-mediated inflammatory signals, such as tumor necrosis factor and lipopolysaccharide [11]. The present study identifies an unexpected role for ASK1 in cell division by regulating spindle orientation and positioning. Mechanistically, our data demonstrate that ASK1 phosphorylates the microtubule-binding protein EB1 and thereby promotes the interaction of EB1 with the plus ends of astral microtubules, leading to the stabilization of astral microtubules, a prerequisite for spindle orientation and positioning. These findings support the notion that astral microtubule stability is important for fine-tuning of spindle movement to ensure its proper alignment during cell division.

Our study reveals S40, T154 and T206 as the residues of EB1 phosphorylated by ASK1. The S40 residue is located in the CH domain, the binding of which to microtubules is well understood [15, 16]. Phosphorylation of S40 may regulate EB1 binding to microtubules by modulating the formation of hydrogen bonds between S40 and nearby amino acids of tubulin. The T154 residue is located in the flexible linker region, and the T206 residue is located in the linker region adjacent to the EBH domain. The linker region is poorly understood due to the lack of structural information. In budding yeast, phosphorylation of a cluster of six serine residues in the linker region of EB1/Bim1p by Aurora-B/Ipl1p impairs its binding to microtubules [17]. In addition, phosphorylation of S155 has been shown to promote EB1 accumulation at microtubule plus ends, while T166 phosphorylation has an opposite effect [18]. These findings suggest that phosphorylation of the linker region may play complicated roles in the regulation of EB1 binding to microtubules.

Several protein kinases and phosphatases have been reported to regulate spindle orientation and positioning through different mechanisms. For example, p21-activated kinase, AMP-activated kinase, protein phosphatase 2A and PP4C contribute to the above processes by modulating astral microtubule formation and/or the localization of various protein complexes at the cell cortex or other cellular sites [19–22]. These findings, together with the role of ASK1 in spindle orientation and positioning, suggest that protein phosphorylation may serve as a critical mechanism regulating various aspects of spindle behavior. In this study, our data demonstrate that the function of ASK1 in spindle orientation/positioning is mediated by its phosphorylation of EB1. However, given that many different factors contribute to the stabilization of astral microtubules and spindle movement [10], it would not be surprising if other proteins were identified in the future mediating ASK1 functions in cell division. Our preliminary study of EB2 and EB3, two other members of the EB family, reveals that while ASK1 interacts with and phosphorylates EB3 similarly to its effect on EB1, it interacts weakly with EB2 and does not phosphorylate EB2 (Supplementary Figure S6). This is consistent with previous findings that the functions of EB1 and EB3 in regulating microtubule dynamics are largely similar or partially redundant, but are strikingly different from the function of EB2 [23, 24]. It will be interesting to investigate in the future the relationship between EB1 and EB3 in mediating the function of ASK1 to control spindle orientation and positioning.

ASK1 has been implicated in the development of several human diseases, such as inflammatory, cardiovascular and neurodegenerative diseases [11]. In addition, ASK1 has been shown to play a role in the pathogenesis of colon, skin, liver and gastric cancers [25–28]. It has been proposed that alteration of ASK1 expression or activity may stimulate tumor development by regulating inflammation, apoptosis or cell proliferation [25–28]. In this study, our data reveal that depletion of ASK1 expression or suppression of its activity leads to spindle orientation/positioning defects in epithelial cells. It is worthy of note that ASK1-deficient mice exhibit no obvious developmental abnormalities and do not spontaneously develop tumors, indicating that spindle orientation/positioning defect alone seems not tumorigenic [29]. However, upon chemical induction, ASK1-deficient mice develop more numerous and larger colon tumors than wild-type mice [26]. Therefore, spindle orientation/positioning defect caused by ASK1 deficiency may synergize with other tumor-associated changes to impair tissue organization and stimulate tumor development.

## Materials and Methods

### Antibodies and chemicals

Antibodies against HA, GST, GFP and Flag (Sigma-Aldrich, St. Louis, MO, USA); phosphoserine and phosphothreonine (Millipore, Billerica, MA, USA); CLIP-170, p150^glued^ and γ-tubulin (Santa Cruz Biotechnology, Santa Cruz, CA, USA); α-tubulin (Abcam, Cambridge, MA, USA); pT845-ASK1 (Cell Signaling Technology, Danvers, MA, USA) and EB1 (BD Biosciences, San Jose, CA, USA) were purchased from the indicated sources. The anti-ASK1 antibody was obtained from Abcam (# ab45178). Horseradish peroxidase-conjugated secondary antibodies were from Amersham Biosciences (Chandler, AZ, USA). Rhodamine- or fluorescein-conjugated secondary antibodies were obtained from Jackson ImmunoResearch Laboratories (West Grove, PA, USA). NQDI-1, paclitaxel and DAPI were from Sigma-Aldrich.

### Generation of the pT206-EB1 antibody

The pT206-EB1 customized antibody was obtained from GL Biochem (Shanghai, China). The phosphopeptide (VNVLKL-pT206-VEDLEKE) was synthesized and conjugated with the carrier protein keyhole limpet hemocyanin (KLH) for immunization. Three 8-week rabbits were immunized with the phosphopeptide for four times. Antibodies were purified with peptide affinity chromatography and verified with enzyme-linked immunosorbent assay and dot blotting assays with good response to the phosphopeptide and no response to the unmodified peptide.

### Plasmids, proteins and siRNAs

Mammalian expression plasmids for Flag-EB1, HA-EB1, GFP-EB1, GST-EB1 and various mutants were described previously [14, 30, 31]. GFP was fused to the C-terminus of EB1. Mammalian expression plasmids for ASK1 and HA-ASK1 were cloned using pcDNA3 and pCMV-HA vectors, and their mutants were generated by PCR and site-directed mutagenesis. Bacterial expression plasmids for GST-EB1, His-EB1 and various mutants were constructed using pGEX-6P3 and pET-28a vectors, and proteins were purified with glutathione sepharose 4B beads (Roche, New York, NY, USA) and nickel-nitrilotriacetic acid beads (Qiagen, Valencia, CA, USA), respectively. *In vitro* transcription and translation were performed by using the TNT quick coupled transcription/translation system (Promega, Fitchburg, WI, USA) following the accompanying protocol. Control siRNA (5′-CGUACGCGGAAUACUUCGA-3′), ASK1 siRNAs (1: 5′-GCACUCCU UCAUCGAGCU-3′; 2: 5′-GGUAUACAUGAGUGGAAUU-3′) and EB1 siRNA (5′-GGAGAAAUGUAAAGACUGA-3′) were synthesized by Ribo Bio (Guangzhou, China).

### Cell culture and transfection

HeLa, 293 T (from the American Type Culture Collection, Manassas, VA, USA) and HeLa-H2B cells (from Eric Griffis, University of Dundee, Dundee, Scotland, UK) were grown in Dulbecco's modified Eagle's medium supplemented with 10% (vol/vol) fetal bovine serum at 37 °C in 5% (vol/vol) CO_2_. All plasmids were transfected into cells with polyethyleneimine (Sigma-Aldrich), and siRNAs were transfected with the lipofectamine RNAiMAX reagent (Invitrogen, Carlsbad, CA, USA).

### Examination of EB1 phosphorylation *in vitro*

ASK1 and ASK1^KD^ were transfected into 293 T cells and immunoprecipitated with anti-ASK1 antibody. Kinase assays were performed at 30 °C for 2.5 h in the kinase reaction buffer (Cell Signaling Technology, Danvers, MA, USA), by using ASK1 or ASK1^KD^ immunoprecipitate and bacterially purified GST-EB1 or His-EB1. To examine EB1 dephosphorylation, GST-EB1 was pulled down from the above reaction mixture with glutathione-coated agarose beads, incubated with λPPase in the dephosphorylation buffer (Cell Signaling Technology) at 35 °C for 2 h and then examined by immunoblotting.

### In-gel digestion and mass spectrometric analysis

GST-EB1 was pulled down from cells with glutathione-coated agarose beads, resolved by SDS/PAGE and stained with Coomassie blue. The GST-EB1 band was then subjected to standard in-gel tryptic digestion. Eluting peptides were loaded onto a Waters Symmetry C18 trapping column (300 μm i.d. 1 cm length) using the Waters NanoAcquity UPLC System, and separated by a linear gradient from 2 to 35% over 40 min at 300 nl min^−1^ through a column packed with 1.7 μm BEH C18 material (Waters, Milford, MA, USA). The Waters Synapt Q-IM-TOF G1 mass spectrometer was operated in high-definition MSE mode, and the data were processed with ProteinLynx Global Server (PLGS v2.4; Waters) to reconstruct MS/MS spectra by combining all masses with a similar retention time. MS/MS spectra were searched against UniProt human sequence database using PLGS. Phosphosite identification had to meet the following criteria: (1) phosphopeptides were identified with a confidence >95% and a PLGS peptide score >6; (2) the mass error of the peptide precursor was below 10 p.p.m.; (3) the MS/MS spectrum was manually inspected to confirm neutral loss and specific fragment ions critical for assigning the modification sites; and (4) phosphorylation sites were assigned consistently in three biological replicates.

### Fluorescence microscopy

Cells were fixed with methanol at −20 °C for 5 min and blocked with 2% bovine serum albumin in phosphate-buffered saline. Cells were then incubated in succession with primary and secondary antibodies followed by staining with DAPI and examined with a TCS SP5 confocal microscope (Leica, Wetzlar, Germany) equipped with LASAF software [32]. The spindle angle and spindle displacement distance were measured as described previously [33, 34]. The intensity and length of EB1 comets were analyzed with the Image J software. For time-lapse microscopy, cells were cultured in a 37 °C chamber, and mitotic progression was recorded with the confocal microscope as described [35].

### Immunoblotting, immunoprecipitation and GST pulldown

Proteins were resolved by SDS/PAGE and transferred onto polyvinylidene difluoride membranes (Millipore). The membranes were blocked and incubated with primary antibodies and then with horseradish peroxidase-conjugated secondary antibodies. Specific proteins were visualized with the enhanced chemiluminescence detection reagent (Millipore). For immunoprecipitation and GST pulldown, cell lysates or purified proteins were incubated with antibody- or glutathione-coated agarose beads or at 4 °C for 2 h. The beads were washed and boiled in the SDS loading buffer, and the proteins were detected by immunoblotting.

### Preparation of polymeric and soluble tubulin fractions from cells

Cells were washed with phosphate-buffered saline, and soluble proteins were then extracted under conditions that prevent microtubule depolymerization (0.1% Triton X-100, 0.1 m MES, pH 6.75, 1 mm MgSO_4_, 2 mm EGTA, 4 m glycerol). The remaining polymeric tubulin fraction containing microtubules and associated proteins was dissolved in 0.5% SDS in 25 mm Tris (pH 6.8). Proteins present in the polymeric and soluble tubulin fractions were then analyzed by immunoblotting.

### *In vitro* microtubule-binding assay

Microtubules were assembled from bovine brain tubulin at 35 °C in the PEMG buffer (100 mm PIPES, 1 mm EGTA, 1 mm MgSO_4_, 1 mm GTP, pH 6.8) for 20 min, and purified His-EB1-WT, -3A, -3D or *in vitro*-phosphorylated His-EB1 was added and incubated for another 30 min. The reaction mixture was then layered over PEMG buffer containing 50% sucrose and 20 μm paclitaxel, and centrifuged at 30 000 r.p.m. for 30 min. The pellet and supernatant fractions were then collected and analyzed by immunoblotting.

### Statistics

Analysis of statistical significance was performed by the Student’s *t*-test for comparison between two groups and by the ANOVA test for multiple comparisons.
