# Supplementary material for: ASK1 controls spindle orientation and positioning by phosphorylating EB1 and stabilizing astral microtubules
Source: Cell Discov. 2016 Oct 4;2:16033–. doi: 10.1038/celldisc.2016.33 (PMC5048754; doi:10.1038/celldisc.2016.33)
Supplement: Supplementary information [file celldisc201633-s1.pdf]

Supplementary information

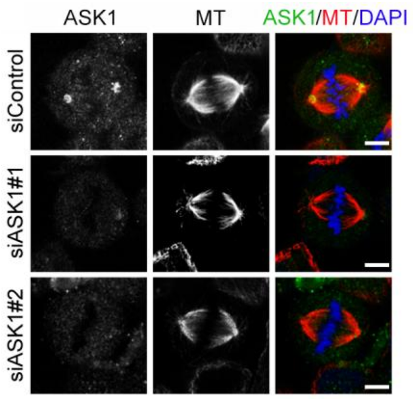

Fig. S1. Knockdown of ASK1 Abolishes Its Localization to Spindle Poles. HeLa cells were transfected with control or ASK1 siRNAs and stained with anti-ASK1 and anti-α-tubulin antibodies and DAPI. Immunofluorescence images of metaphase cells were then taken. Scale bars, 5 μm.

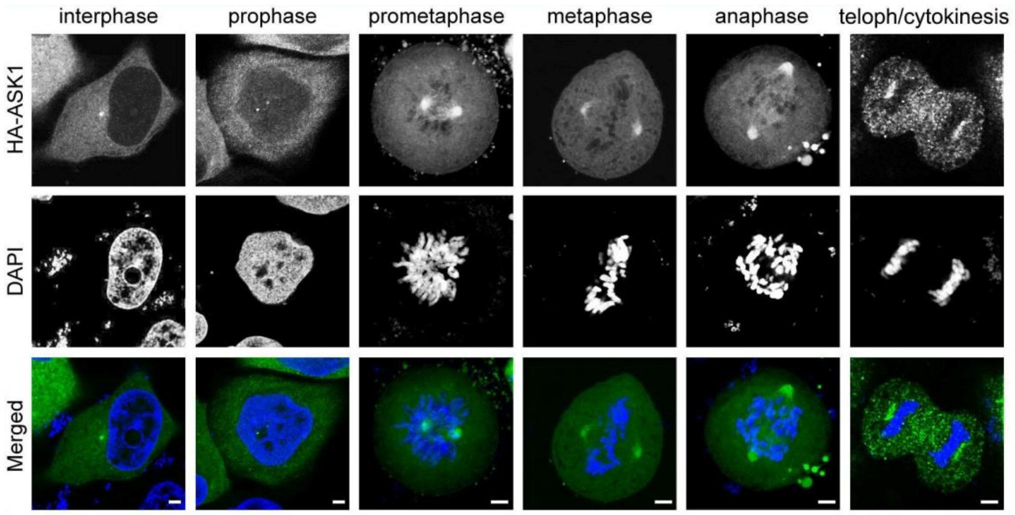

Fig. S2. HA-ASK1 Localizes to Spindle Poles. HeLa cells were transfected with HA-ASK1 and stained with anti-HA antibody (green) and DAPI (blue). Immunofluorescence images of cells at different mitotic phases were then taken. Scale bars, 5 μm.

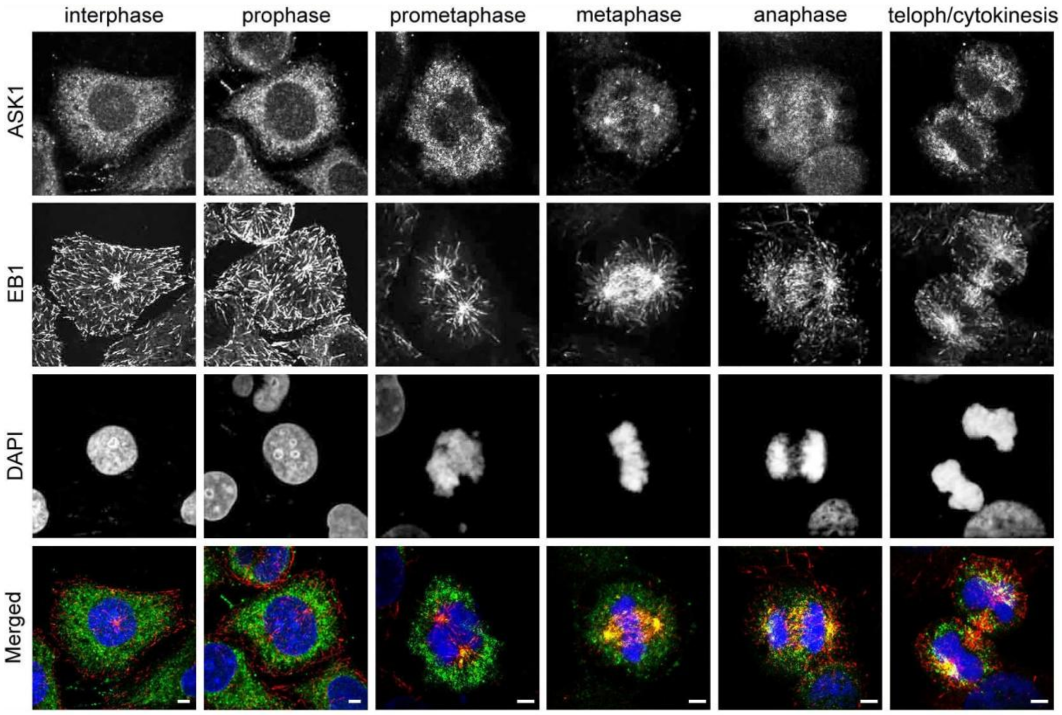

Fig. S3. Endogenous ASK1 and EB1 Colocalize at Spindle Poles. HeLa cells were stained with anti-ASK1 (green) and anti-EB1 (red) antibodies and DAPI (blue). Immunofluorescence images of mitotic cells were then taken. Scale bars, 5 µm.

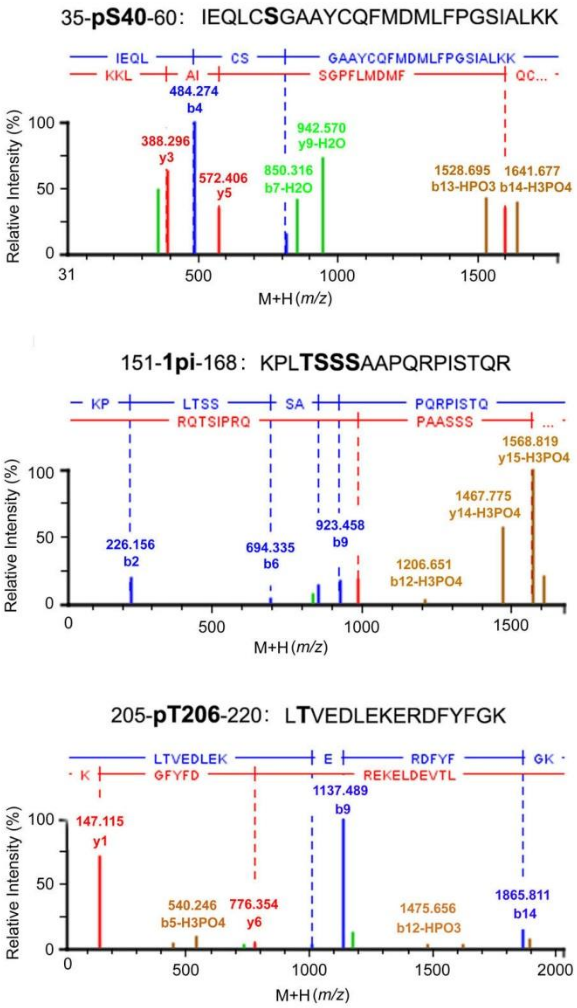

Fig. S4. Mass Spectrometric Profiles of GST-EB1 Phosphopeptides.

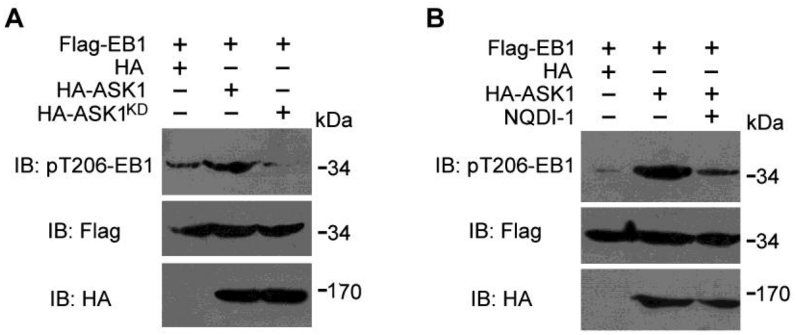

Fig. S5. Confirmation of T206 as a Site Phosphorylated by ASK1 in Cells. (A) Immunoblots showing the level of EB1 phosphorylation at T206 in 293T cells transfected with Flag-EB1 and HA, HA-ASK1, or HA-ASK1^{KD}. (B) Immunoblots showing the level of EB1 phosphorylation at T206 in 293T cells transfected with Flag-EB1 and HA or HA-ASK1 and treated with the ASK1 inhibitor NQDI-1 (15 μM).

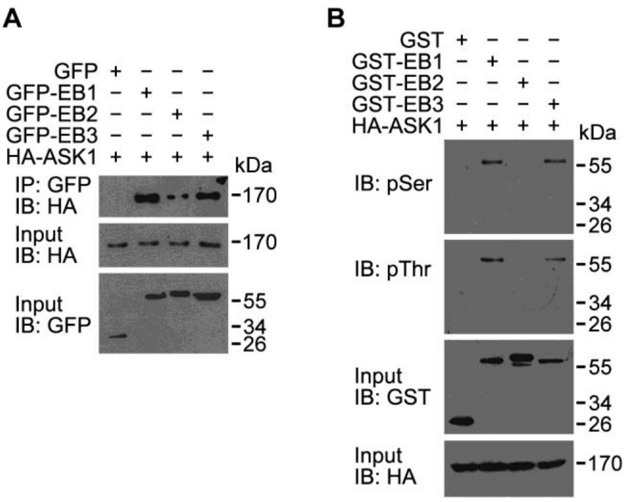

Fig. S6. Comparison of the Interactions of EB Family Members with ASK1 and Their Phosphorylation by ASK1. (A) Immunoprecipitation and immunoblotting showing that HA-ASK1 interacts with GFP-EB1 and GFP-EB3, but not GFP-EB2. (B) Kinase assays were performed by using HA-ASK1 immunoprecipitate from 293T cells, with bacterially purified GST-EB1, -EB2, or -EB3 as substrate. The reaction mixture was then subjected to immunoblotting with pSer, pThr, GST, and HA antibodies.
